# Characterization of the postsynaptic protein neurogranin in paired cerebrospinal fluid and plasma samples from Alzheimer’s disease patients and healthy controls

**DOI:** 10.1186/s13195-015-0124-3

**Published:** 2015-07-01

**Authors:** Hlin Kvartsberg, Erik Portelius, Ulf Andreasson, Gunnar Brinkmalm, Konstantin Hellwig, Natalia Lelental, Johannes Kornhuber, Oskar Hansson, Lennart Minthon, Philipp Spitzer, Juan M Maler, Henrik Zetterberg, Kaj Blennow, Piotr Lewczuk

**Affiliations:** Department of Psychiatry and Neurochemistry, Institute of Neuroscience and Physiology, The Sahlgrenska Academy, University of Gothenburg, House V3/SU Mölndal, SE-431 80 Mölndal, Sweden; AlzeCure Foundation, Karolinska Institutet Science Park, Hälsovägen 7, SE-141 57 Huddinge, Sweden; Department of Psychiatry and Psychotherapy, Universitätsklinikum Erlangen and Friedrich-Alexander-Universität Erlangen-Nürnberg, Schwabachanlage 6, 91054 Erlangen, Germany; Department of Clinical Sciences Malmö, Memory Clinic, Clinical Memory Research Unit, Faculty of Medicine, Lund University, Malmö, Klinikgatan 22, SE-222 42 Lund, Sweden; Department of Molecular Neuroscience, UCL Institute of Neurology, Queen Square 588, WC1N 3BG London, UK

## Abstract

**Introduction:**

Synaptic dysfunction and degeneration are central events in Alzheimer’s disease (AD) pathophysiology that are thought to occur early in disease progression. Synaptic pathology may be studied by examining protein biomarkers specific for different synaptic elements. We recently showed that the dendritic protein neurogranin (Ng), including the endogenous Ng peptide 48 to 76 (Ng_48–76_), is markedly increased in cerebrospinal fluid (CSF) in AD and that Ng_48–76_ is the dominant peptide in human brain tissue. The aim of this study was to characterize Ng in plasma and CSF using mass spectrometry and to investigate the performance of plasma Ng as an AD biomarker.

**Methods:**

Paired plasma and CSF samples from patients with AD (n = 25) and healthy controls (n = 20) were analyzed in parallel using an immunoassay developed in-house on the Meso Scale Discovery platform and hybrid immunoaffinity-mass spectrometry (HI-MS). A second plasma material from patients with AD (n = 13) and healthy controls (n = 17) was also analyzed with HI-MS. High-resolution mass spectrometry was used for identification of endogenous plasma Ng peptides.

**Results:**

Ng in human plasma is present as several endogenous peptides. Of the 16 endogenous Ng peptides identified, seven were unique for plasma and not detectable in CSF. However, Ng_48–76_ was not present in plasma. CSF Ng was significantly increased in AD compared with controls (*P* < 0.0001), whereas the plasma Ng levels were similar between the groups in both studies. Plasma and CSF Ng levels showed no correlation. CSF Ng was stable during storage at −20°C for up to 2 days, and no *de novo* generation of peptides were detected.

**Conclusions:**

For the first time, to our knowledge, we have identified several endogenous Ng peptides in human plasma. In agreement with previous studies, we show that CSF Ng is significantly increased in AD as compared with healthy controls. The origin of Ng in plasma and its possible use as a biomarker need to be further investigated. The results suggest that CSF Ng, in particular Ng_48–76_, might reflect the neurodegenerative processes within the brain, indicating a role for Ng as a potential novel clinical biomarker for synaptic function in AD.

**Electronic supplementary material:**

The online version of this article (doi:10.1186/s13195-015-0124-3) contains supplementary material, which is available to authorized users.

## Introduction

Alzheimer’s disease (AD) is a neurodegenerative disease characterized by neuropathological changes in the brain, including extracellular amyloid-β (Aβ) deposits called plaques as well as neurofibrillary tangles consisting of hyperphosphorylated tau protein (p-tau) [1,2]. Lower cerebrospinal fluid (CSF) levels of the 42–amino acid–long Aβ peptide Aβ_1–42_, reflecting the plaque pathology, together with increased concentrations of total tau (t-tau) and p-tau, reflecting neurodegeneration and tangle pathology, respectively, are today considered the core CSF biomarkers for AD [3].

A central event in AD pathology is synaptic dysfunction and degeneration [4]. Synaptic loss correlates with cognitive decline and is more associated with the degree of dementia compared to plaques and tangles [5-8], especially within certain areas of the brain, such as the hippocampus [7,9]. Furthermore, synaptic loss has been identified as an early event in the disease progression, as well as the underlying cause of the progressive cognitive deterioration as the disease advances [4]. Thus, synaptic markers are promising tools for early AD diagnosis. Moreover, such biomarkers could also be used to monitor disease progression and play an important role in the evaluation of novel disease modifying therapeutics.

Neurogranin (Ng) is a postsynaptic protein with a critical role in long-term potentiation, where it regulates the levels of calmodulin in response to intracellular calcium levels after neuronal excitation [10-12]. In the brain, Ng is localized to dendritic spines of neurons within associative cortical areas, including the hippocampus, amygdala and cerebral cortex [11,13]. Studies on rodents have shown that both the Ng mRNA and total protein levels are age-dependently decreased in several regions of the brain, including the hippocampus [14]. In addition, *in situ* hybridization studies on the human brain have shown that the Ng mRNA is selectively translocated to dendrites and that such translocation is impaired in the cortex of patients with AD [15].

Recently, we generated three monoclonal anti-Ng antibodies with epitopes near the C-terminal part of the protein [16]. By immunoprecipitation using the antibodies Ng2 and Ng3, followed by high-resolution mass spectrometry (MS), we identified several endogenous Ng peptides in both brain tissue and CSF. A third monoclonal antibody, Ng7, was used as a capturing antibody in an enzyme-linked immunosorbent assay (ELISA) developed in-house for measuring CSF Ng. In three independent cohorts, we showed increased CSF Ng levels in AD CSF as compared with healthy controls. Using hybrid finity MS (HI-MS), we also showed that the endogenous Ng peptide 48 to 76 (Ng_48–76_) was significantly increased in AD CSF as compared with controls [16].

If a synaptic marker reflecting the ongoing synaptic degeneration in the central nervous system (CNS) could be identified in blood or plasma, it would be a very valuable clinical tool because CSF sampling is considered to be a somewhat more invasive procedure. In addition, simple and repeated sampling would also be made possible, allowing for dynamic changes to be measured over time at a regular clinic. Therefore, in this study of paired plasma and CSF samples, we aimed to first characterize Ng in plasma using MS and then investigate whether plasma Ng is a marker of synaptic degeneration in AD.

Using MS, we show—for the first time, to our knowledge—that Ng is expressed as a variety of endogenous Ng peptides in human plasma. Although plasma Ng was not found to differ between AD patients and healthy controls, we replicated the previous finding of increased CSF Ng in the AD group using an in-house assay developed on the Meso Scale Discovery (MSD) platform. Finally, we also show that CSF Ng is stable during storage and that no peptides are generated *de novo*. The endogenous peptide Ng_48–76_, which in our previous study was found to be significantly increased in both patients with AD and individuals with prodromal AD, was not found in plasma. The findings presented in the present study imply that CSF Ng might reflect the ongoing neurodegenerative processes within the CNS, indicating a role for Ng, and in particular Ng_48–76_, as a potential novel clinical biomarker for synaptic function in AD.

## Methods

Anonymized plasma samples collected in the routine workflow at the Neurochemistry Laboratory at Sahlgrenska University Hospital, Mölndal, Sweden, were used for method development, as approved by the ethics committee at the University of Gothenburg.

### Patients in paired cerebrospinal fluid and plasma study

The paired CSF and plasma study included 25 AD patients (mean age: 76 ± 8 years) and 20 control subjects without dementia (mean age: 54 ± 14 years) (see Table 1 for demographics and biomarker characteristics). The AD group consisted of patients with probable AD with a high level of evidence of an AD pathophysiologic process and patients with mild cognitive impairment with a high likelihood of an underlying AD process. In the rest of this article, this group is called *AD*. The patients were recruited in Erlangen, Germany, and each underwent a thorough clinical investigation, including a medical history as well as a physical, neurological and psychiatric examinations. The diagnosis of AD was made according to the National Institute of Neurological and Communicative Disorders and Stroke-Alzheimer’s Disease and Related Disorders association (NINCDS-ADRDA) criteria [17]. No patient with AD had a family history of dementia suggestive of autosomal dominant AD. The control group consisted of patients with other neuropsychiatric conditions without dementia. The clinical diagnoses (including neuroimaging and neuropsychology) were combined with the results of the following CSF biomarkers: Aβ_1–42_, Aβ_1–40_ (to calculate the Aβ_42/40_ concentration ratio), t-tau and p-tau181. The subjects were included in the study only if the clinical, neuroimaging and neuropsychological diagnoses were in accordance with the neurochemical findings as described elsewhere [18]. There were no patients included in the study with a completely negative AD biomarker pattern. Control subjects had all biomarkers within normal ranges. CSF t-tau, p-tau, Aβ_1–40_ and Aβ_1–42_ concentrations were determined using established ELISA methods as described elsewhere [18]. All analyses were performed at the Universitätsklinikum Erlangen. The study was conducted according to the provisions of the Declaration of Helsinki. All subjects gave their written informed consent for the use of their clinical data for research purposes, as approved by the ethics committee of the Universitätsklinikum Erlangen.

**Table 1:** **Demographic and clinical characteristics of subjects included in the study 1 and 2** ^**a**^

	**Control**	**AD**
**Study 1 (paired plasma + CSF)**	**n = 20**	**n = 25**
Sex, n, female/male (% female)	12/8 (60)	14/11 (56)
Age at LP, yr	54 (41 to 63)	76 (71 to 78)^b^
CSF Aβ_1–42_, pg/ml	1,399 (1189 to 1702)	709 (588 to 897)^b^
CSF Aβ_1–42_ **/**Aβ_1–40_ ratio	0.094 (0.079 to 0.104)	0.033 (0.028 to 0.042)^b^
CSF t-tau, pg/ml	185 (136 to 254)	580 (425 to 655)^b^
CSF p-tau, pg/ml	35 (27 to 45)	89 (78 to 105 to 126)^b^
CSF Ng, pg/ml (MSD)	291 (251 to 438)	620 (521 to 818)^b^
Plasma Ng, pg/ml (MSD)	47,451 (21,904 to 90,320)	36,525 (25,324 to 57,715)
Plasma, pg/ml (HI-MS)	21,698 (13,401 to 106,954)	25,644 (17,262 to 53,900)
**Study 2 (plasma)**	**n = 17**	**n = 13**
Sex, n, female/male (% female)	9/17 (53)	9/13 (69)
Age at LP, yr	58 (53 to 68)	78 (69 to 80)^b^
CSF Aβ_1–42_, pg/ml	875 (580 to 940)	330 (268 to 415)^b^
CSF t-tau, pg/ml	300 (210 to 348)	690 (605 to 980)^b^
CSF p-tau, pg/ml	48 (40 to 61)	110 (77 to 160)^b^

^a^Aβ, Amyloid-β; AD, Alzheimer’s disease; CSF, Cerebrospinal fluid; HI-MS, Hybrid immunoaffinity-mass spectrometry; LP, lumbar puncture; MSD, Meso Scale Discovery; Ng, Neurogranin; p-tau, Phosphorylated tau; t-tau, Total tau. The values presented are median (interquartile range). ^b^ *P* < 0.001 vs. controls.

### Patients and plasma samples included in the verification study

Plasma samples were collected from 13 patients with AD (mean age: 78 ± 7 years) and 17 individuals without dementia (controls; mean age: 58 ± 9 years) at the Memory Clinic, Skåne University Hospital, Sweden (see Table 1 for full biomarker characteristics and demographics). The individuals underwent brain imaging, routine laboratory testing and neurological, psychiatric and cognitive examinations. Patients diagnosed with AD fulfilled the dementia criteria of the *Diagnostic and Statistical Manual of Mental Disorders, Third Edition, Revision* [19], and the criteria for probable AD as defined by NINCDS-ADRDA [17]. The control individuals without dementia experienced subjective cognitive symptoms at baseline, but thorough clinical investigation as well as clinical follow-up revealed that the patients were not affected by a dementia disorder or a neurological disease. CSF t-tau, p-tau and Aβ_1–42_ concentrations were determined using established ELISA methods as described elsewhere [16]. All analyses were performed at the Clinical Neurochemistry Laboratory at the Sahlgrenska University Hospital, Mölndal, Sweden. All individuals gave their informed consent to participate in research before their samples were stored in a biobank. A passive consent procedure was then used whereby consent for retrospective use of banked samples and basic data was assumed if individuals did not actively retract permission, as instructed in local press advertisements. This study procedure was approved by the local ethics committee at Lund University, Sweden.

### Meso Scale Discovery neurogranin assay

The monoclonal mouse antibody Ng7, with an epitope including amino acids 52 to 65 [16], was used as a capturing antibody and was coated on QUICKPLEX 96-well plates (Meso Scale Discovery (MSD), Rockville, MD, USA) at a final concentration of 2.0 μg/ml (40 μl/well) in phosphate-buffered saline (PBS), followed by incubation overnight at room temperature (RT). Before being coated with the capturing antibody, the plates were washed once with 150 μl of PBS. After the plates were washed with 300 μl of PBS containing 0.05% Tween 20 (PBS-Tween) four times for 30 seconds each time, the remaining protein-binding sites were blocked with 5% MSD Blocker A solution (150 μl/well; Meso Scale Discovery) for 1 hour at RT and subsequently washed again with 300 μl of PBS-Tween four times for 30 seconds each time. Then the full-length Ng calibrators with concentrations ranging between 31.3 and 4,000 pg/ml (1:2 dilution; CSF samples) or 31.3 to 23,000 pg/ml (1:3 dilution; plasma samples), blanks and samples (50 μl/well) were incubated in duplicate together with the detecting antibody, polyclonal Ng anti-rabbit antibody (ab23570; Upstate Biotechnology, Lake Placid, NY, USA) diluted 1:20,000 in 0.1% bovine serum albumin (BSA) in PBS-Tween overnight at RT (50 μl/well). Plasma samples were diluted 1:40 in PBS. After washing four times for 30 seconds each time in PBS-Tween, a MSD SULFO-TAG goat anti-rabbit antibody (25 μl/well; Meso Scale Discovery), concentration 0.5 μg/ml diluted in 0.1% BSA in PBS-Tween, was added and incubated for 2 hours at RT while being shaken at 700 rpm. After washing with 300 μl of PBS-Tween four times for 30 seconds each time, 150 μl of 2× MSD Read Buffer T with surfactant (Meso Scale Discovery) were used for immediate reading with a QUICKPLEX SQ 120 reader (Meso Scale Discovery). A fitted four-parameter logistic model with relative weighting (1/*y*^2^) was used as the calibration curve, and the blank was included as zero concentration of Ng. All samples were analyzed without knowledge of clinical diagnosis.

### Immunoprecipitation/matrix-assisted laser desorption/ionization time-of-flight/time-of-flight analysis

To minimize nonspecific binding of other proteins during the immunoprecipitation, all plasma samples were pretreated twice with 20 μl of Novex Dynabeads Protein G (Life Technologies, Carlsbad, CA, USA) per milliliter of plasma and incubated for 1 hour at RT before the beads were magnetically removed as described previously, with some modifications [20]. HI-MS analysis of Ng peptides was performed as described elsewhere [16]. In brief, 4 μg of the monoclonal anti-Ng antibodies Ng2 and Ng3 were separately added to 25 μl of Dynabeads M-280 sheep anti-mouse immunoglobulin G (Life Technologies) and cross-linked as previously described [21]. Beads coated with Ng2 and Ng3 were used for immunoprecipitation of plasma to which *n*-octyl-β-d-glucopyranoside (10%, end concentration 0.1%; Fluka Chemie, Buchs, Switzerland) had been added. The beads and sample were transferred to a KingFisher magnetic particle processor (polypropylene tubes; Thermo Scientific, Waltham, MA, USA) for automatic washing and elution of the Ng peptides and protein. Eluted Ng was collected and dried in a vacuum centrifuge and redissolved in 5 μl of 0.1% formic acid (FA) in 20% acetonitrile and subsequently analyzed using an ultrafleXtreme matrix-assisted laser desorption/ionization (MALDI) time-of-flight/time-of-flight (TOF/TOF) MS (Bruker Daltonics, Bremen, Germany). All solvents used were of high-performance liquid chromatography (HPLC) quality.

In both the paired cohort and validation study where MALDI-TOF/TOF MS was used, the custom Ng peptide RKKIKSGERGRKGPGPGGPGGAGVARGGAGGGP (corresponding to Ng_43–75_), with glycine labeled with double ^13^C (theoretical molecular mass: 3,011 Da; CASLO, Lyngby, Denmark), was added to the plasma prior to sample preparation and was used as an internal standard. Samples were analyzed blinded (that is, without knowledge of clinical diagnosis).

### High-resolution tandem mass spectrometry

The identities of the Ng peptides were confirmed using a top-down approach as previously described [22], but with updated instrumentation. Briefly, analysis was performed using nanoflow liquid chromatography (Dionex UltiMate 3000; Thermo Scientific) coupled to an electrospray ionization Q Exactive Hybrid Quadrupole Orbitrap tandem mass spectrometer (Thermo Scientific). An Acclaim PepMap 100 C8 trap column (length: 20 mm; inner diameter: 75 μm; particle size: 3 μm; pore size: 100 Å (Thermo Scientific)) was used for online desalting, and a reversed-phase Acclaim PepMap rapid separation liquid chromatography column (length: 500 mm, inner diameter: 75 μm; particle size: 2 μm; pore size: 100 Å (Thermo Scientific)) was used for high-resolution separation. Mobile phases were 0.1% aqueous FA (v/v) (A) and 0.1% FA in 84% acetonitrile in water (v/v) (B). The separation was performed at a flow rate of 150 nl/min by applying a linear gradient of 3% to 40% B for 50 minutes. A column oven was used and set to 40°C. All solvents used were of HPLC quality. The Q Exactive system was used in positive ion mode to acquire full-scan spectra in the 300 to 1,200 mass-to-charge (*m*/*z*) ratio range at a resolution setting of 70,000. Subsequent tandem mass spectra (MS/MS) were produced using the higher-energy collisional dissociation fragmentation cell and acquired at the same resolution setting. An inclusion list was employed to ensure that we obtained MS/MS data of the compounds of interest. Mass spectral raw files were then charge-deconvoluted by using Mascot Distiller software (Matrix Science, Boston, MA, USA) and subsequently submitted to a Mascot database search (Matrix Science).

### Degradation of neurogranin in plasma and cerebrospinal fluid at room temperature

Plasma and CSF samples were depleted of native Ng by three consecutive immunoprecipitations before full-length recombinant Ng (1,000 pg/ml) was added to the samples, followed by 24-hour incubation at RT. After the incubation, samples were analyzed by HI-MS to screen for degradation products from the full-length Ng protein in the form of newly formed Ng peptides.

### Cerebrospinal fluid neurogranin storage stability assessment

Pooled CSF samples with or without a stabilizing agent (sodium azide at a final concentration of 0.1% in samples), were kept at RT, 4°C or −20°C for 1 to 7 days before they were placed in long-term storage at −80°C pending analysis. As a reference sample, a freshly prepared aliquot, with or without stabilizer, was stored at −80°C on the day of the preparation (that is, day 0). All other samples were normalized against the Ng content of the respective reference sample for either group. Ng content was analyzed by MSD.

### Statistical analysis

For statistical analysis, Prism 6 for Windows software (GraphPad Software, La Jolla, CA, USA) was used. Because biomarker values were skewed, nonparametric tests were used. Differences between groups were assessed using the Mann–Whitney *U* test, and significance was determined at *P* < 0.05. Correlations were determined using Spearman’s correlation.

## Results

### Characterization of plasma neurogranin

**Figure 1:**
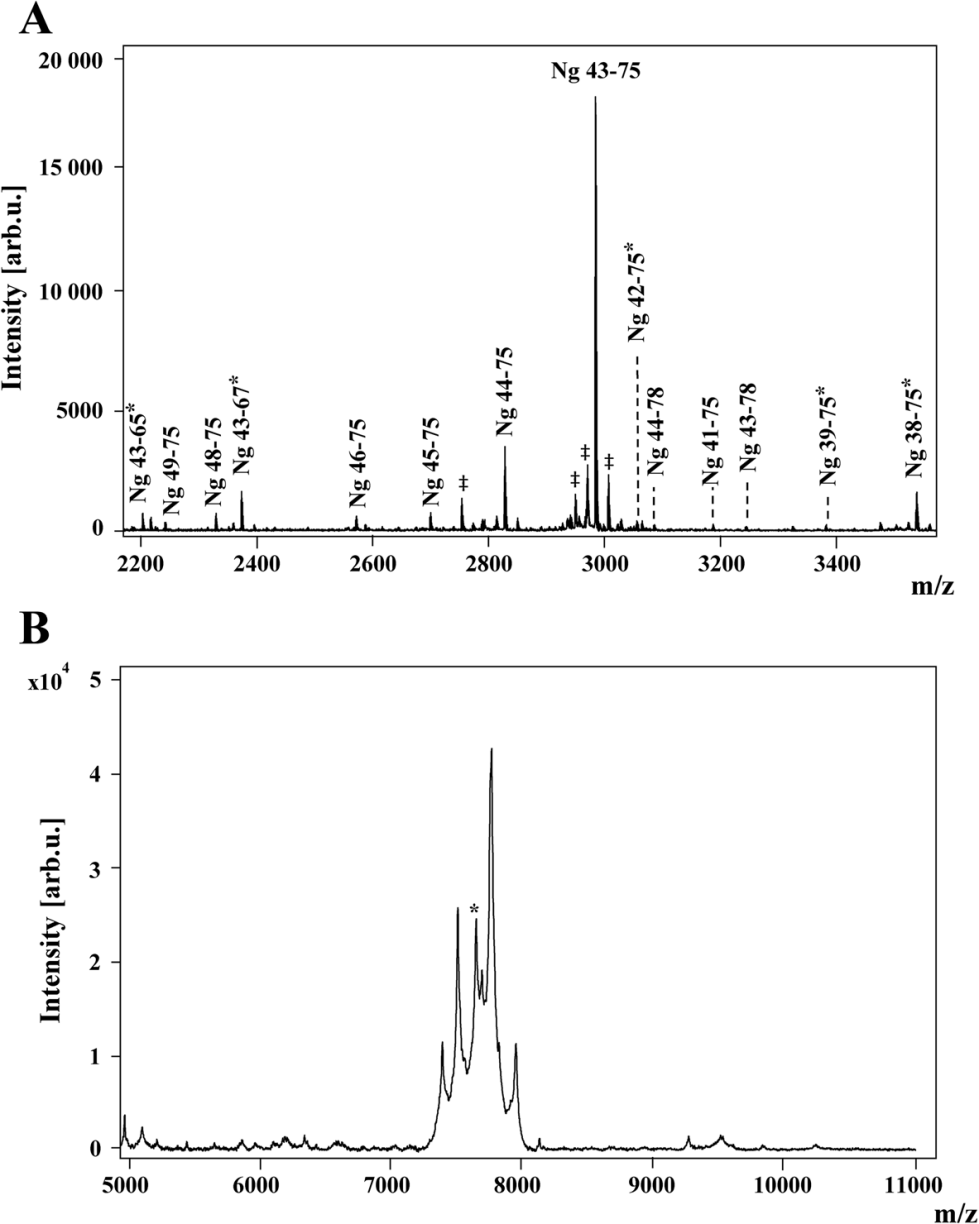
Hybrid immunoaffinity-mass spectrometry characterization of plasma neurogranin. **(A)** Hybrid immunoaffinity-mass spectrometry analysis of human plasma repeatedly detected several short C-terminal peptides. *Peptides found only in plasma and not in cerebrospinal fluid or brain tissue. ^‡^Nonspecific binding of other plasma proteins to magnetic beads. **(B)** A cluster of peaks roughly corresponding to the mass of full-length neurogranin (Ng) is present in plasma. *Peak corresponding to the theoretical mass of full-length Ng (7,618 Da). *m*/*z*, Mass-to-charge ratio.

Plasma Ng was characterized using MALDI-TOF/TOF and high-resolution MS/MS. Several short C-terminal peptides were repeatedly detected by MALDI-TOF/TOF, and 16 of these were independently verified and identified by high-resolution MS/MS (Figure 1A and Table 2). Of these, seven had not previously been detected in brain tissue or CSF [16]. A summary of all Ng peptides that have been identified using MS in plasma, CSF and brain tissue can be found in Additional file 1: Table S1. A cluster of peaks at the *m*/*z* ratio corresponding roughly to full-length Ng (theoretical mass: 7,618 Da) was detected (Figure 1B), indicating that the protein most likely is subjected to several posttranslational modifications; however, these have yet to be identified.

**Table 2:** **Identified plasma neurogranin peptides by high-resolution mass spectrometry** ^**a**^

**Calculated mass (Da)**	**Theoretical mass (Da)**	**Deviation (ppm)**	**Confirmed sequence**	**Number of y-ions**	**Number of b-ions**
1,899.98	1,899.98	−1.3	52 to 75^b^	1	11
2,203.24	2,203.24	1.7	43 to 65^b^	1	14
2,217.24	2,217.25	−1.8	44 to 67^b^	2	8
2,242.14	2,242.14	−0.1	49 to 75	5	12
2,329.18	2,329.17	1.0	48 to 75	10	20
2,373.34	2,373.35	−1.0	43 to 67^b^	0	6
2,570.36	2,570.35	1.8	46 to 75	10	17
2,698.45	2,698.45	0.1	45 to 75	12	19
2,826.54	2,826.54	−2.4	44 to 75	19	21
2,982.64	2,982.64	−1.7	43 to 75	21	8
3,053.69	3,053.68	2.9	42 to 75^b^	18	6
3,085.61	3,085.62	−4.3	44 to 78	6	12
3,184.73	3,184.72	2.7	41 to 75	14	4
3,241.72	3,241.73	−1.6	43 to 78	19	13
3,378.81	3,378.80	1.8	39 to 75^b^	8	2
3,534.91	3,534.90	1.7	38 to 75^b^	11	10

^a^Mass deviation is displayed in parts per million (ppm). ^b^Peptides found only in plasma and not in cerebrospinal fluid or brain tissue.

### Clinical validation of paired plasma and cerebrospinal fluid samples

**Figure 2:**
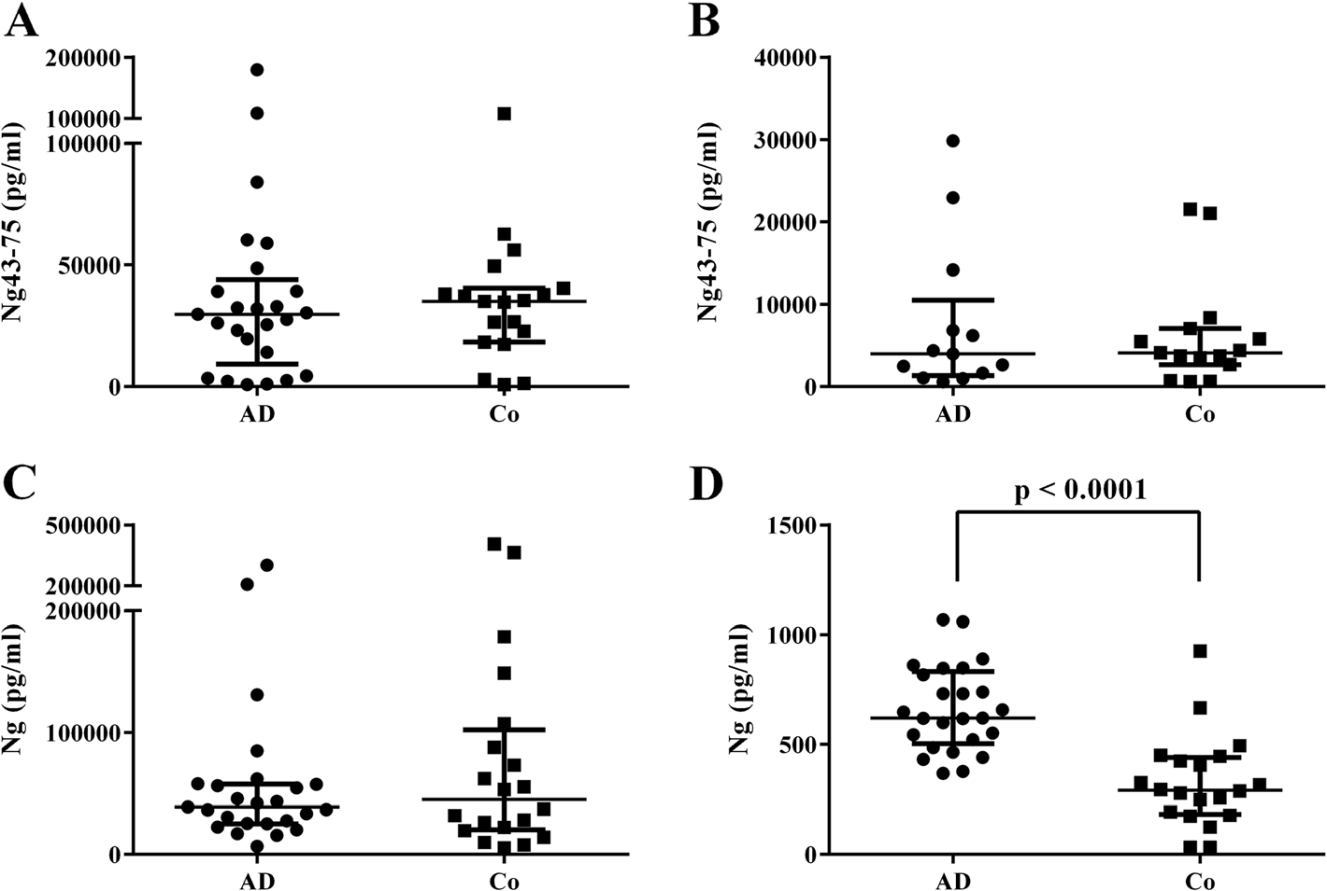
Scatterplots displaying the results from hybrid immunoaffinity-mass spectrometry and Meso Scale Discovery analysis of plasma and cerebrospinal fluid samples. **(A)** Scatterplot of plasma concentrations of the most abundant neurogranin (Ng) peptide 43 to 75 (Ng_43–75_), in samples from the paired cohort obtained by hybrid immunoaffinity-mass spectrometry (HI-MS). **(B)** Scatterplot of plasma Ng_43–75_ concentrations in samples from the verification study obtained by HI-MS. **(C)** Scatterplot of plasma Ng levels obtained by Meso Scale Discovery (MSD) in samples from the paired cohort. **(D)** Scatterplot of cerebrospinal fluid Ng levels obtained by MSD. The data presented are medians and interquartile ranges. AD, Alzheimer’s disease; Co, Control.

Paired plasma and CSF samples from patients with AD and controls were analyzed for Ng levels to investigate the potential of plasma Ng as a novel biomarker. Plasma samples were analyzed in parallel by using an MSD platform assay and HI-MS targeting both the shorter endogenous peptides and full-length Ng. Using HI-MS, several plasma Ng peptides were reliably detected and quantified, but none were found to differ significantly between the groups. Concentrations of the most abundant plasma Ng peptide, Ng_43–75_, which was present in all samples, are displayed in Figure 2A. This finding was replicated by HI-MS analysis in a second independent plasma material, where, once again, none of the peptides differed between patients with AD and healthy controls. Concentrations of Ng_43–75_ obtained in the validation study are displayed in Figure 2B. The peaks at the mass range of full-length Ng (Figure 1B) were also investigated, but no significant difference was obtained between patients with AD and healthy controls (data not shown). In agreement with this, MSD analysis of plasma Ng did not reveal any quantitative differences between the groups (Figure 2C).

In contrast, MSD analysis of the CSF samples showed a marked increase of Ng in patients with AD (*P* < 0.0001) (Figure 2D). As the control group was significantly younger than the AD group in both studies (see Table 1), we also tested for a correlation between CSF Ng and age, but no such association was found in the paired cohort (AD group: *P* = 0.667, *r*_s_ = −0.1; control group: *P* = 0.11, *r*_s_ = −0.37) or in the validation study (AD group: *P* = 0.258, *r*_s_ = 0.34; control group: *P* = 0.77, *r*_s_ = −0.08). No correlation between CSF and plasma Ng was found in the paired cohort (*P* = 0.83, *r*_s_ = 0.0334). Owing to the large CSF volume needed for HI-MS analysis (1 ml), the CSF samples were analyzed only by MSD.

### Neurogranin storage stability and degradation in cerebrospinal fluid and plasma

**Figure 3:**
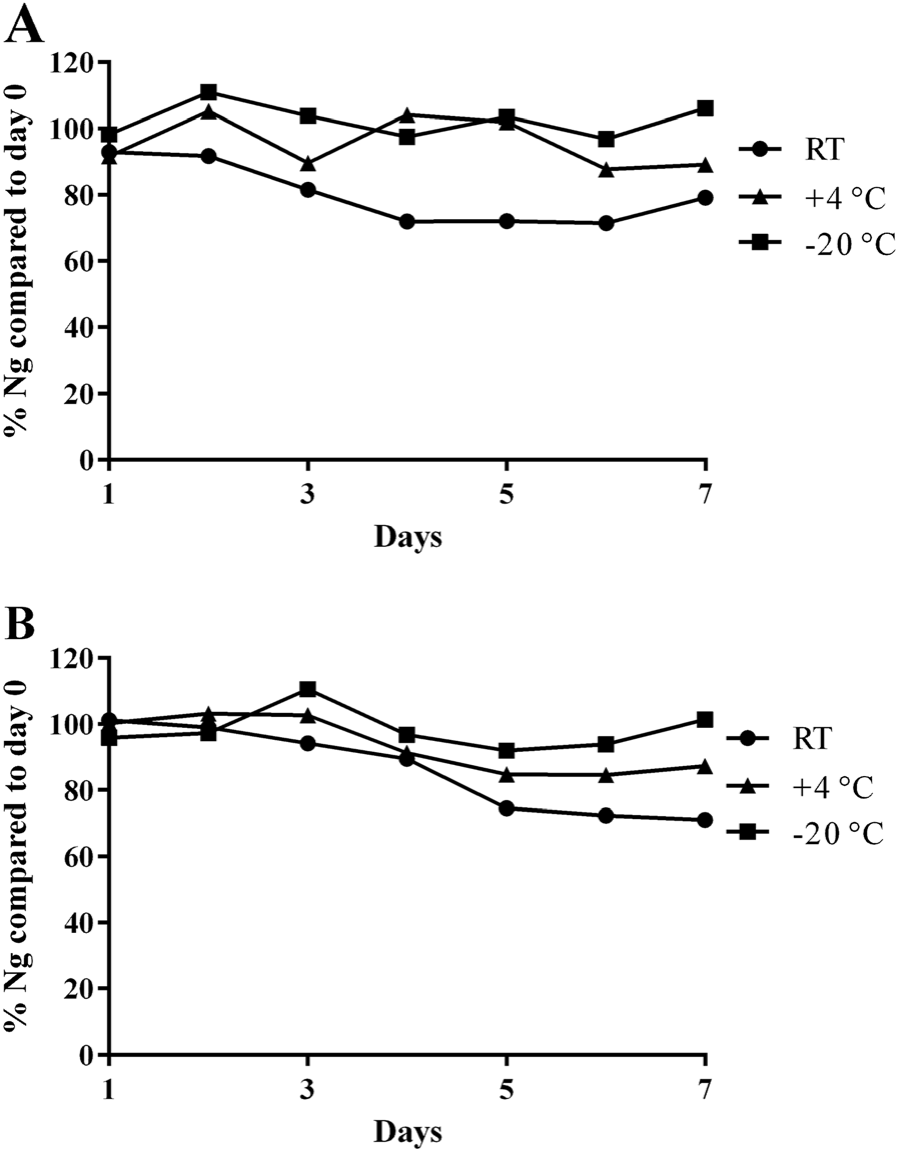
Analysis of cerebrospinal fluid neurogranin storage stability. Normalized cerebrospinal fluid neurogranin (Ng) concentrations in samples without **(A)** or with **(B)** the addition of a stabilizing agent. Samples were kept at room temperature (RT), 4°C or −20°C for between 1 and 7 days before transfer to storage at −80°C pending analysis. Samples were normalized to day 0, with or without stabilizer.

With the purpose of investigating the stability of CSF Ng during storage, CSF was kept at RT, 4°C or −20°C with or without the addition of a stabilizer for between 1 and 7 days before it was stored at −80°C pending analysis. The samples were analyzed using the MSD Ng assay, and all values were normalized against a reference sample, which was stored at −80°C on the day of preparation. The CSF Ng levels were stable at RT for 2 days, after which the concentration dropped to around 70% compared with the day of preparation. Samples kept at either 4°C or −20°C were both stable for all time points investigated (Figure 3A). Similar results were obtained when a stabilizing agent was added (Figure 3B).

To address the question whether any degradation of Ng occurs in CSF and plasma, CSF and plasma samples that had been depleted of all native Ng by immunoprecipitation using Ng2 and Ng3 were spiked with full-length Ng (1,000 pg/ml) and incubated at RT for 24 hours. The samples were then analyzed for newly generated Ng peptides using HI-MS. In CSF, no Ng peptides generated *de novo* could be detected in the spiked samples after the incubation (data not shown). In contrast, 14 Ng peptides were detected in the spiked plasma samples, indicating that Ng is degraded in plasma (Additional file 2: Table S2).

## Discussion

In this study, we characterized the Ng content in plasma and investigated the potential of plasma Ng as a biomarker for AD. We also addressed the stability of CSF Ng during storage and investigated any potential degradation of Ng in both CSF and plasma.

In total, we identified 16 endogenous Ng peptides in plasma. We also show that full-length Ng most likely is present, but mostly in posttranslationally modified forms. Of the endogenous plasma peptides, seven had not been detected previously in either brain tissue or CSF [16]. Thus, they seem to be plasma-specific. As we have now performed HI-MS analysis of Ng peptides in more than 75 plasma samples in total and the plasma-specific peptides have been present in all of these, we can conclude that these are consistently found in plasma and are thus most likely not caused by the preanalytical handling,

In a previous study, we showed increased CSF Ng levels in AD using ELISA [16]. In the same study, using MS, we also found that, of the endogenous Ng peptides examined, Ng_48–76_ showed the most pronounced increase in AD CSF compared with controls. Importantly, Ng_48–76_ was also found to be the dominant peptide in AD brain tissue [16], but this peptide was not found in plasma (Table 2). These data suggest that this particular peptide most likely is brain-specific. Furthermore, there are also peptides that are unique for plasma as well as for CSF and brain tissue (Additional file 1: Table S1). Thus, Ng peptides detected in CSF may specifically reflect ongoing neurodegenerative processes within the brain and are most likely not influenced by Ng peptides produced in the periphery, indicating that they may have valid clinical relevance. Further supporting CSF Ng as an AD marker are the discoveries that the plasma Ng levels did not differ between AD and controls (Figure 2A, B and C) and that there was a lack of correlation between CSF and plasma Ng content.

In a series of experiments designed to test the stability of Ng in plasma, we show that recombinant full-length protein incubated in plasma, depleted of native Ng, is metabolized into a variety of fragments (Additional file 2: Table S2), probably by some of the proteases present in plasma. Five of the detected degradation fragments most likely correspond to endogenous peptides that were identified in native plasma samples, and, in addition to those, we also detected a number of peaks that most likely correspond to other Ng peptides that have not been seen in their native counterparts (Additional file 2: Table S2). Also, four of the Ng peptides found only in plasma were not generated after incubation of full-length Ng in Ng-depleted plasma, thus indicating that they are not an artefact of sample handling. This indicates that there are several enzymes present in plasma that are capable of cleaving Ng. As the peptide patterns of native plasma Ng and degraded full-length Ng differ, this might point toward differences regarding the enzymes in freeze–thawed plasma samples and enzymes in plasma *in vivo* with regard to activity and/or specificity. These differences could be due to a number of different factors, such as pH or storage stability. However, a characterization of the active enzymes in plasma after −80°C storage that are capable of cleaving full-length Ng to generate the peptides observed in such samples is beyond the scope of this article.

Ng_48–76_ was neither naturally occurring (Table 2) nor generated after incubation of full-length Ng in the plasma samples investigated (Additional file 2: Table S2). Therefore, it is unlikely that this specific peptide is produced in the periphery. These findings indicate that the enzyme responsible for the formation of Ng_48–76_ is present only in the brain or at least is not active in either CSF or plasma. This hypothesis is further supported by the fact that, though there are three Ng peptides in CSF that end with amino acid 76 (Additional file 1: Table S1), neither these endogenous peptides (Table 2) nor fragments generated from recombinant full-length protein (Additional file 2: Table S2) were found in plasma. The enzymes responsible for the different cleavages of Ng remains to be identified.

Compared with CSF, plasma Ng concentration in paired samples was around 100 times higher (Figure 2C and D). Studies have shown that Ng is expressed in the periphery in the spleen, bone marrow, platelets and lung tissue [11], which may contribute to the high concentration in plasma.

Limitations of both studies are their small sample size and that healthy control subjects were significantly younger than the AD patient groups (*P* < 0.001) (Table 1). However, considering that no difference in plasma Ng was found between the groups in either cohort and that increased CSF Ng in AD has been shown before in age-matched groups [16], we believe that the age difference between the groups did not affect the results of this study. Also, we did not find any correlation between age and Ng concentration in either plasma or CSF.

## Conclusions

The present study shows that Ng is present in plasma at high concentrations, both as posttranslationally modified full-length protein and as a number of endogenous peptides. The previously reported difference in Ng levels in CSF between controls and AD patients is a consistent finding that is not reflected in the plasma. The most important discoveries are that there is no degradation of full-length Ng or *de novo* formation of peptides in CSF and that CSF Ng is stable for storage at – 20°C, as well as that there are no native plasma Ng peptides ending with amino acid 76. This suggests that CSF Ng, especially Ng_48–76_, might reflect ongoing neurodegenerative processes within the brain, indicating a role for Ng as a potential novel clinical biomarker for synaptic function in AD.

## Additional files

Additional 1: Table S1. Summary of all Ng peptides identified by MS/MS in human brain tissue, CSF and plasma. Microsoft Excel 97–2003 worksheet (.xls). A complete list of all endogenous Ng peptides identified by MS/MS in human brain tissue, CSF and plasma. Additional 2: Table S2. Newly formed Ng peptides in plasma incubated with recombinant full-length Ng. Microsoft Excel 97–2003 worksheet (.xls). Plasma samples were depleted of native Ng by three consecutive immunoprecipitations before full-length recombinant Ng (1,000 pg/ml) was added to samples, followed by 24-hour incubation at RT. After the incubation, the samples were analyzed by HI-MS to screen for newly formed Ng peptides.

## Abbreviations

Aβ: Amyloid-β
AD: Alzheimer’s disease
BSA: Bovine serum albumin
CNS: Central nervous system
CSF: Cerebrospinal fluid
ELISA: Enzyme-linked immunosorbent assay
FA: Formic acid
HI-MS: Hybrid immunoaffinity-mass spectrometry
HPLC: High-performance liquid chromatography
MALDI: Matrix-assisted laser desorption/ionization
MS: Mass spectrometry
MS/MS: Tandem mass spectrometry
*m*/*z*: Mass-to-charge ratio
Ng: Neurogranin
NINCDS-ADRDA: National Institute of Neurological and Communicative Disorders and Stroke/Alzheimer’s Disease and Related Disorders Association
PBS: Phosphate-buffered saline
ppm: Parts per million
p-tau: Phosphorylated tau
RT: Room temperature
TOF/TOF: Time-of-flight/time-of-flight
t-tau: Total tau
